# Relevance of Kidney‐Metabolic Multimorbidity Pattern to Metabolic Health and Mortality Among Elderly Inpatients in China

**DOI:** 10.1002/fsn3.71612

**Published:** 2026-03-23

**Authors:** Lan Xu, Mengjing Wang, Guoyou Qin, Qiang Shao, Hong Huang, Huaizhou You, Jing Chen

**Affiliations:** ^1^ Division of Geriatrics Huashan Hospital, Fudan University Shanghai China; ^2^ Division of Nephrology Huashan Hospital, Fudan University Shanghai China; ^3^ National Clinical Research Center for Aging and Medicine Huashan Hospital, Fudan University Shanghai China; ^4^ Department of Biostatistics, School of Public Health Fudan University Shanghai China; ^5^ Information Center of Huashan Hospital Fudan University Shanghai China

**Keywords:** “kidney‐metabolic” multimorbidity pattern, aging, metabolic health, mortality, nutrition

## Abstract

As populations age globally, multimorbidity has emerged as a significant challenge. Chronic kidney disease (CKD) is a common aging related condition whose contribution to mortality is rising, yet CKD associated multimorbidity are seldom reported. This study aims to investigate the prevalence and patterns of multimorbidity, with a specific focus on the CKD associated multimorbidity pattern and its impact on metabolic health and mortality. We conducted a cross‐sectional analysis using a large dataset from Huashan hospital at Fudan University, Shanghai, China. Data were collected for patients aged over 60 years between January 1, 2013, and January 1, 2019. In total, 48 chronic diseases were included and multimorbidity was defined as the co‐occurrence of two or more of these 48 diseases in a single patient. Our database included 163,626 elderly inpatients with a mean age of 69.82 ± 7.75 years. The number of morbidities was positively associated with mortality. 80.7% of patients with CKD had other comorbidities. Factor analysis revealed four multimorbidity patterns in the elderly inpatients, named as “Kidney‐metabolic”, “Cerebro‐vascular”, “Cardio‐pulmonary” and “Thyroid‐digestive” pattern. The “Kidney‐metabolic” pattern, characterized by CKD, Hypertension, Diabetes, and Lipid disorders, exhibited the highest prevalence at 13.3%. All four multimorbidity patterns were significantly positively associated with increased mortality. However, the impact of the “Kidney‐metabolic” pattern on mortality was the only one that significantly increased with age. Our results suggest that, “Kidney‐metabolic” pattern, the most prevalent among the elderly, significantly highlighted intertwined nutrition and metabolic‐related conditions and represented predominant combined risk factors for metabolic health.

## Graphical abstract

In this study, we established a large‐scale research database named as “Elderly Multimorbidity Database”. We introduced the novel identification of the “Kidney‐metabolic” multimorbidity pattern. The “Kidney‐metabolic” pattern, being the most prevalent in our study, significantly increases mortality risk, particularly in the oldest age groups.

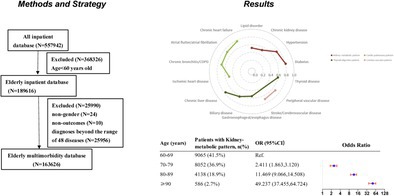

## 1 Introduction

With the progress of global aging, China now grapples with the challenge of accommodating and supporting its expanding aging population. In 2020, 18% of its population were over age 60, and 2.5% were over age 80, projected to rise to 39% and 10%, respectively, by 2050 (Fang et al. 2025). The accelerated aging is accompanied by a dramatic increase in the prevalence of multiple chronic diseases. Aging‐related chronic diseases have emerged as a significant public health challenge in China (Zhang et al. 2018).

The core physiological changes of aging are characterized by various metabolic disorders caused by a decline in the body's homeostatic regulatory capacity. Due to its high metabolic demands, the kidney is particularly susceptible to aging, making it one of the organs most significantly affected (Khan et al. 2017). Chronic kidney disease (CKD) is defined as a reduction in kidney function or structural damage (or both) present for more than 3 months, with the high global prevalence of about 14.2% (13.4%–15.2%) (Collaborators 2025). The kidney engages in numerous vital interactions with other body organsand commonly accompanied by other chronic diseases such as hypertension, diabetes, cardiovascular diseases, stroke, osteoporosis, dementia, and frailty in the elderly (Wang et al. 2021; Zimmerman et al. 2020). Such coexistence of multiple chronic diseases in an individual is named as multimorbidity (Barnett et al. 2012). The tendency toward certain chronic diseases to cluster has been identified as multimorbidity patterns or clusters (Prados‐Torres et al. 2014). Previous studies found the common multimorbidity patterns among elderly adults include the cardio‐pulmonary, vascular‐metabolic, cognitive‐emotional, and skeletal‐muscle patterns, with CKD seldom included (Ioakeim‐Skoufa et al. 2022, 2020; Yao et al. 2020). Studies focused on multimorbidity pattern related to CKD are fewer in elderly (Hu et al. 2024).

Multimorbidity and patterns not only lead to more pronounced consequences, including higher mortality, increased disability and a lower quality of life (Barnett et al. 2012), but also changes the traditional treatment model and poses significant challenges to public health decision‐making and management. Particularly among the elderly, malnutrition occurs, especially protein‐energy malnutrition, put forward severe and urgent response demands for the public health system (Yee‐Moon Wang et al. 2023). Elderly patients with CKD accompanied by metabolic‐related diseases will not only suffer from the continuous impact of multiple metabolic disorders but also fall into an extremely higher risk of malnutrition in a complex state of multimorbidity. Elevated fasting blood glucose, high lipid level, and elevated systolic blood pressure are all major risk factors for life years with disabilities related to CKD (Collaborators 2025). Our focus should move away from management of specific nutrients and toward the broader perspective of whole diets and dietary patterns.

Research on multimorbidity in China began relatively late. Large‐scale studies on multimorbidity among inpatients from tertiary hospitals are scarce in China (Teng et al. 2019). As the first tertiary comprehensive hospital to be awarded Joint Commission International (JCI) certification in China, Huashan Hospital's medical data includes patients spanning diverse regions of the country. Therefore, our study aims to investigate the characteristics of multimorbidity and multimorbidity patterns in elderly inpatients from China's comprehensive hospital, particularly focusing on the CKD‐associated multimorbidity pattern and its impact on metabolic health and mortality.

## 2 Methods and Materials

### 2.1 Study Design and Participants

Our study conducted a cross‐sectional analysis of a large single‐center dataset maintained by Huashan hospital of Fudan University, Shanghai, China. The dataset comprised information including age, gender, diagnoses, and mortality data, extracted from electronic medical records of the hospital spanning January 1, 2013, to January 1, 2019. To ensure privacy, the data were meticulously anonymized, thereby eliminating any possibility of identifying individuals. The study adhered to the Declaration of Helsinki and received approval from the local Medical Ethics Committee at Huashan Hospital, Fudan University, Shanghai, China (Approval number: KY2019‐362).

### 2.2 Data Governance and Standardization of Disease Diagnose

This study established a multidisciplinary team including the Information Center, Medical Department, Research Department, clinical physicians, and epidemiology experts to jointly carry out the following data governance and standardization of disease diagnosis. The team interfaced with the hospital's Clinical Data Repository (CDR), Hospital Information System (HIS), Electronic Medical Record (EMR), Laboratory Information Management System (LIS), and other IT platforms to conduct data collection, data cleaning, and data integration. From these data sources, general demographic data and hospitalization information (disease diagnosis, hospitalization date, discharge date, transfer date, time of death, and death diagnosis) were extracted for inpatients over 60 years old.

Diagnoses were encoded based on the International Classification of Diseases, Tenth Revision (ICD‐10). Permanent chronic diseases were characterized by irreversible pathological changes or the need for rehabilitation or long‐term care (Corsonello et al. 2020; Laux et al. 2008). Specifically, CKD diagnoses were reassessed by nephrologists in accordance with the criteria established by the Kidney Disease: Improving Global Outcomes (KDIGO) (Stevens et al. 2013). Adhering to the hierarchical organization of the original ICD‐10 coding, we subsequently categorized all 1310 chronic disease diagnoses into 85 broader categories. Ultimately, based on the requirements of our study and insights from prior research (Barnett et al. 2012; Corsonello et al. 2020; Zheng et al. 2021), 48 chronic disease diagnoses with a prevalence exceeding 0.1% were selected. The database we established was named as “Elderly Multimorbidity Database”. Table S1 details the 48 diseases included in the study of multimorbidity. Multimorbidity was defined as a patient having two or more of the selected 48 diseases.

## 3 Statistical Analysis

We calculated the prevalence of multimorbidity among all participants. Categorical variables were reported as counts and percentages. All participants with available variables of interest were included, and missing data were not imputed. The means were analyzed using Analysis of Variance (ANOVA), and proportions using the Chi‐square test.

Patterns of multimorbidity were subjected to exploratory factor analysis (EFA) (Gu et al. 2017; Ioakeim‐Skoufa et al. 2020; Nunes, Camargo‐Figuera, et al. 2016). Due to the unique characteristics of Cancer and its impact on mortality, Cancer was excluded from the factor analysis. To mitigate statistical noise and potential spurious findings, diseases with a prevalence of less than 1% were excluded, and 14 diseases identified as core chronic conditions in the elderly were selected (see Supplement Table S2). Each chronic disease was coded as a dichotomous variable (0 = no disease, 1 = presence of the disease). The Kaiser‐Meyer‐Olkin (KMO) method and Bartlett's Test of Sphericity were employed to assess data adequacy. Factors were retained based on eigenvalues greater than 1 and the scree plot's shape. An oblique rotation (Oblimin) was applied to the factor‐loading matrices for enhanced interpretability. A chronic disease was deemed to be influenced by a given factor if its loading exceeded ±0.5 in that factor (pattern). If the factor loading exceeded ±0.5 across multiple factors, the disease was categorized under the factor with the highest loading. Patients were classified into a specific pattern if they had three or more diseases with factor loadings greater than ±0.5 (Schaefer et al. 2010). Patients were considered to exhibit a pattern if they had fewer than three diseases, each with a factor loading greater than ±0.5, concurrently. The prevalence of multimorbidity patterns within each age and sex group was identified. Associations between patterns and mortality were analyzed using multivariate logistic regression models. Analyses were conducted using SPSS V.26.0 software (IBM, West Grove, Pennsylvania, USA), with statistical significance set at *p* < 0.05 for two‐tailed tests.

## 4 Results

### 4.1 Characteristics and Prevalence of Multimorbidity in the Study Participants

Our analysis was conducted on data drawn from a population of 557,942, excluding patients younger than 60 years old, those with incomplete information on gender or outcomes, and those with diagnoses beyond the scope of our study, ultimately selecting 163,626 individuals aged over 60 years old with 48 chronic diseases (Figure S1). In this study, our participants were from 33 of the 34 provinces, municipalities, and autonomous regions across China (Figure S2 and Table S3).

The mean age of the total population was 69.82 ± 7.75 years. Of these, 55% were males. Demographic characteristics and multimorbidity details are depicted in Table 1. The overall prevalence of multimorbidity reached 38.8% (95% CI: 38.6%–39.0%), with the average number of morbidities being 1.74 ± 1.28. Multimorbidity was observed in 40% (95% CI: 36.9%–37.6%) of males and 37.3% (95% CI: 39.7%–40.3%) of females. There was a significant increase in the prevalence of multimorbidity and the number of chronic diseases with age (Table 1). Among the age groups of 60–69, 70–79, 80–89, and ≥ 90, the prevalence of multimorbidity was 33.2%, 42.3%, 52.9%, and 67.4%, respectively (*p* < 0.001).

**TABLE 1:** Characteristics of demography and multimorbidity.

	Patients *n* (%)	Mean number of morbidities (SD)*	Percentage with multimorbidity^a^ (95% CI)%
Total	163,626 (100%)	1.74 ± 1.28	38.8% (38.6–39.0)
Sex			
Male	90,019 (55.0%)	1.80 ± 1.37	40.0% (39.7–40.3)
Female	73,607 (44.9%)	1.67 ± 1.16	37.3% (36.9–37.6)
Age (years)			
60–69	90,683 (55.4%)	1.56 ± 1.00	33.1% (32.8–33.5)
70–79	51,462 (31.4%)	1.79 ± 1.23	42.3% (41.8–42.7)
80–89	19,374 (11.8%)	2.27 ± 1.80	52.9% (52.2–53.6)
≥ 90	2101 (1.27%)	3.41 ± 2.66	67.4% (53.3–81.5)
Number of chronic diseases			
1	100,149 (61.2%)	··	··
2	33,256 (20.3%)	··	··
3	16,715 (10.2%)	··	··
4	7327 (4.5%)	··	··
5	2921 (1.8%)	··	··
6	1327 (0.8%)	··	··
7	726 (0.4%)	··	··
≥ 8	1205 (0.7%)	··	··

* Differences between means within each variable differed significantly *p* < 0.001 (*t*‐test for independent samples for sex; one‐way ANOVA for age‐group and deprivation).

^a^ Differences between categories within each variable differed significantly *p* < 0.001 (*χ* ^2^ test for 2 × *n* tables).

### 4.2 Prevalence and co‐Occurrence of Chronic Diseases Among Multimorbid Elderly Participants

Among the total participants, cancer, hypertension, diabetes, cerebrovascular disease, and ischemic heart disease constituted the most prevalent chronic conditions, with prevalence rates of 33.3%, 23.1%, 12.1%, 10.9%, and 9.9% respectively. The overall prevalence of CKD in the population stood at 6.0% (Table S1).

**FIGURE 1:**
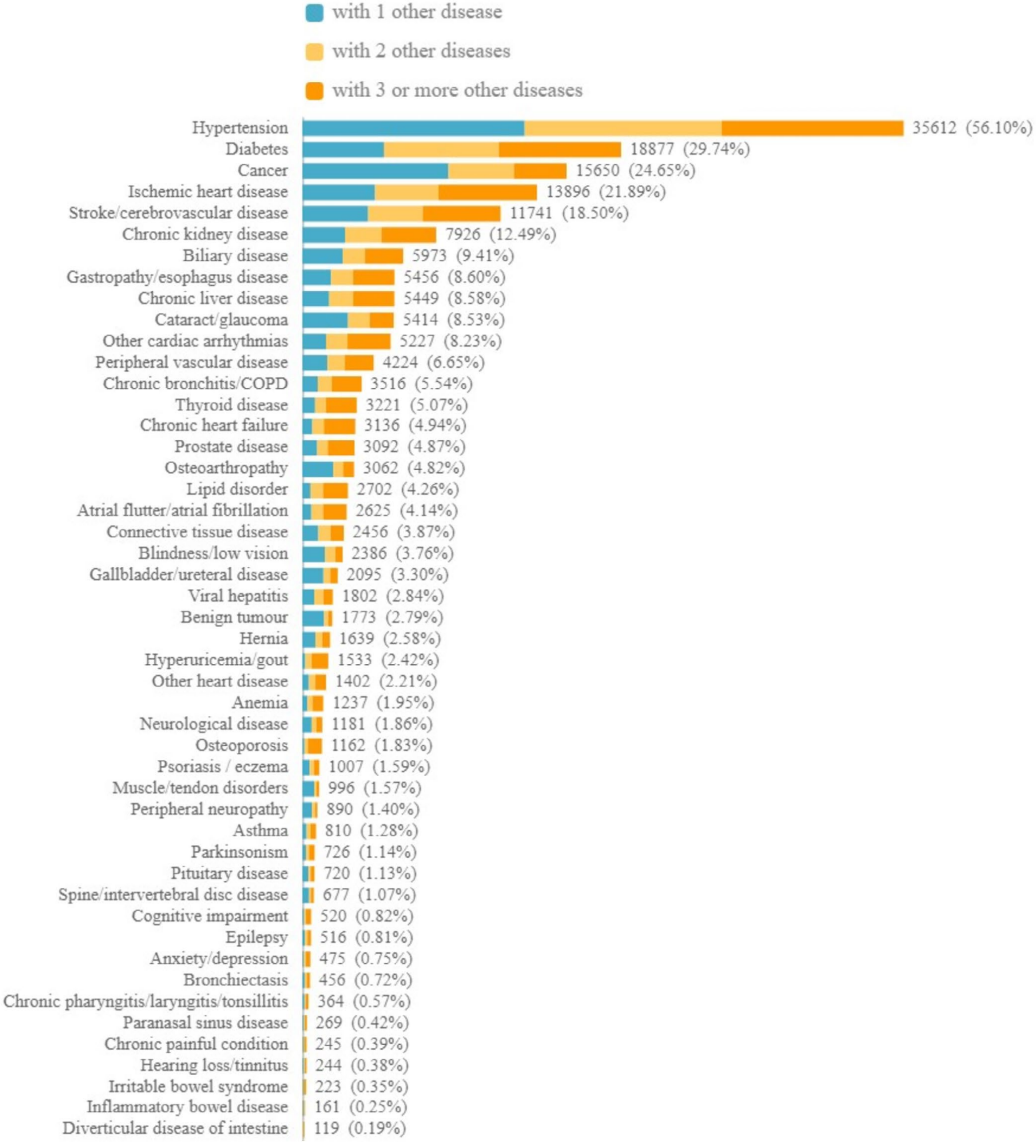
The distribution of chronic diseases in participants with multimorbidities.

Among the 63,477 (38.8%) participants with multimorbidity, the frequent chronic diseases that co‐existed with other diseases were hypertension, diabetes, cancer, cerebrovascular disease, ischemic heart disease, and CKD (Figure 1 and Table S2).

### 4.3 Chronic Conditions co‐Occurring With CKD in Elderly Inpatients

We further analyzed the chronic conditions that frequently co‐occurred with CKD among 9824 (6.0%) participants. Of these, 1898 (19.3%) patients presented with CKD exclusively, while 7962 (80.7%) exhibited additional morbidities. Hypertension, diabetes, bladder/ureteral conditions, cancer, and cardiovascular disease were identified as the chronic conditions most commonly co‐occurring with CKD among elderly inpatients (Table S4).

### 4.4 Identification and Prevalence of Four Distinct Multimorbidity Patterns in Elderly Patients Through Factor Analysis

**FIGURE 2:**
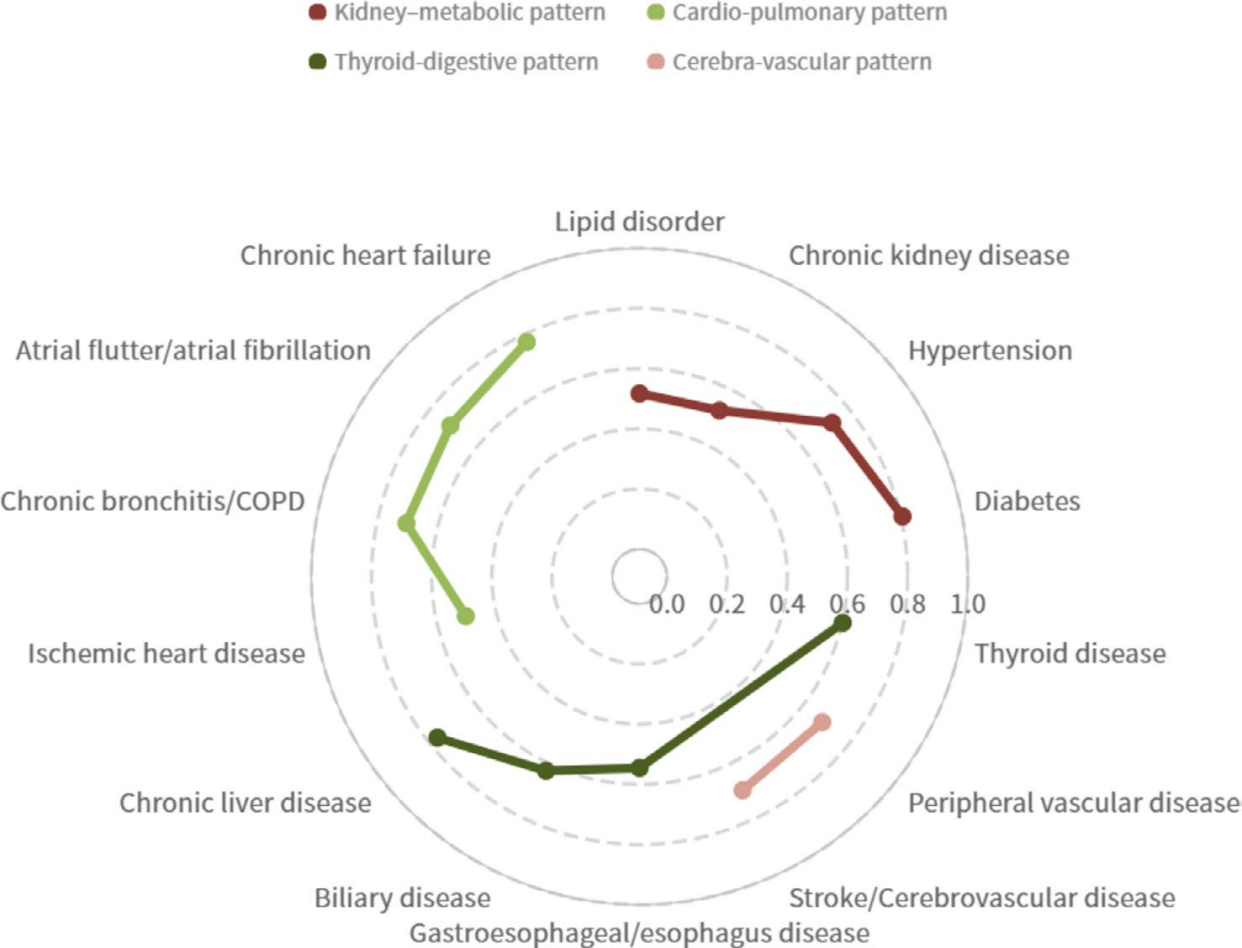
Four patterns identified by factor analysis.

Factor analysis revealed four distinct patterns among our study participants, with a cumulative contribution rate of 56.51% after the extraction of the four factors. A KMO value of 0.7409 and Bartlett's Test of Sphericity (*p* < 0.001) suggested moderate adequacy for sampling in the factor analysis. The “Kidney‐metabolic” pattern, characterized by an eigenvalue of 3.79 and high factor loadings for CKD, hypertension, diabetes, and lipid disorder, emerged as a prominent pattern. Characterized by an eigenvalue of 1.71 and high factor loadings for ischemic heart diseases, chronic heart disease, atrial flutter/atrial fibrillation, and bronchitis/COPD, this pattern was identified as the “Cardio‐pulmonary” pattern. The “Thyroid‐digestive” pattern, with an eigenvalue of 1.32, displayed high factor loadings for chronic liver disease, biliary disease, gastropathy/esophagus disease, and thyroid disease. Named the “Cerebro‐vascular” pattern, the fourth pattern (eigenvalue = 1.08) demonstrated high factor loadings for stroke/cerebrovascular disease and peripheral vascular disease (Figure 2 and Table 2).

**TABLE 2:** The factor loadings of chronic diseases in the corresponding factors.

Chronic diseases	Factor (Multimorbidity Pattern)			
	1	2	3	4
Diabetes	**0.8056**	−0.0533	0.0459	0.0575
Hypertension	**0.7278**	0.2422	−0.0301	0.1104
Chronic kidney disease	**0.5210**	0.1052	0.1735	0.3806
Lipid disorder	**0.5169**	−0.1551	0.3996	0.1825
Chronic heart failure	0.1766	**0.7736**	−0.0114	−0.0998
Atrial flutter/Atrial fibrillation	−0.0227	**0.7134**	−0.0208	0.2289
Bronchitis/COPD	−0.065	**0.7038**	0.1382	0.0122
Ischemic heart disease	0.4844	**0.5014**	−0.1164	−0.0469
Chronic liver disease	0.1319	−0.0467	**0.7682**	0.0025
Biliary disease	−0.1493	0.1296	**0.6255**	−0.277
Thyroid disease	0.1862	−0.0672	**0.5015**	0.145
Gastroesophageal disease	−0.1828	0.4037	**0.5451**	0.1218
Stroke/Cerevoscular disease	0.116	0.2029	−0.1026	**0.6978**
Peripheral vascular disease	0.0129	−0.0837	0.1471	**0.6856**

*Note:* KMO: 0.7409; The factor loading ≥ 0.5 have been highlighted in bold.

Abbreviation: COPD, Chronic obstructive pulmonary disease.

A total of 30.2% of participants (49,472 cases) exhibited one or more multimorbidity patterns. The “Kidney‐metabolic” pattern emerged as the most prevalent pattern at 13.3%, succeeded by the “Cerebro‐vascular” pattern at 12.4%, the “Cardio‐pulmonary” pattern at 2.3%, and the “Thyroid‐digestive” pattern at 2.1%. Prevalence rates for all four patterns showed significant increases with age (*p* < 0.001). With the exception of the “Thyroid‐digestive” pattern, other patterns were notably more prevalent among males than females (*p* < 0.001) (Table 3).

**TABLE 3:** The number and proportions of participants assigned to each multimorbidity pattern.

	*N*	Kidney‐metabolic pattern	Cerebro‐vascular pattern	Cardio‐pulmonary pattern	Thyroid‐digestive pattern	*p* value
Overall, *n* (%)	163,626	21,841 (13.4%)	20,354 (12.4%)	3792 (2.3%)	3485 (2.1%)	
Gender, *n* (%)						
Female	73,607	9408 (12.8%)	7795 (10.6%)	1259 (1.7%)	1693 (2.3%)	< 0.001
Male	90,019	12,433 (13.8%)	12,559 (14.0%)	2533 (2.8%)	1792 (2.0%)	
Age, *n* (%)						
60–69	90,683	9065 (10.0%)	11,024 (12.2%)	585 (0.7%)	1889 (2.1%)	< 0.001
70–79	51,462	8052 (15.7%)	6265 (12.2%)	1251 (2.4%)	997 (1.9%)	
80–89	19,374	4138 (21.4%)	2638 (13.6%)	1451 (7.5%)	488 (2.5%)	
≥ 90	2107	586 (27.8%)	427 (20.3%)	505 (25.0%)	111 (5.3%)	
Outcome, *n* (%)						
Alive	47,016	20,974 (96.0%)	19,580 (96.2%)	3155 (83.2%)	3307 (94.9%)	< 0.001
Dead	2456	867 (4.0%)	774 (3.8%)	637 (16.8%)	178 (5.1%)	

### 4.5 Mortality Risk and Age‐Related Variations in Multimorbidity Patterns

**FIGURE 3:**
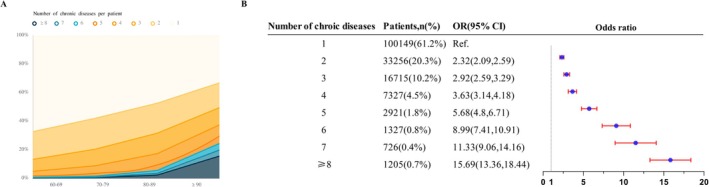
Number of chronic diseases in each age group and its association with mortality by logistic regression.

**FIGURE 4:**
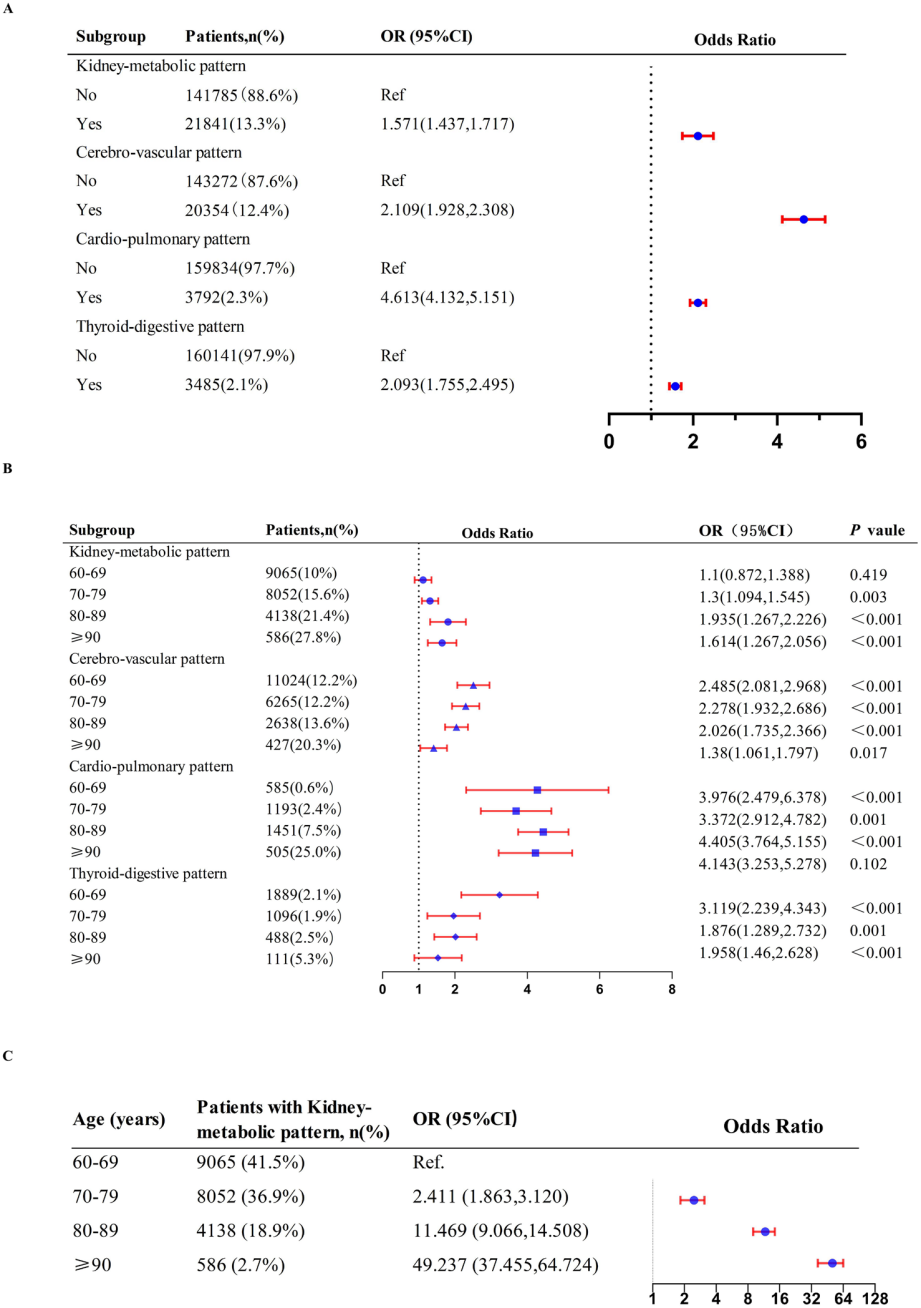
Associations of multimorbidity patterns with mortality by logistic regression.

In this study, the overall mortality rate amounted to 1.8% (867 cases). Mortality risk significantly escalates with an increase in the number of chronic diseases (Figure 3). All identified patterns were positively associated with increased mortality risk. Patients within the “Cardio‐pulmonary” pattern exhibited the highest mortality rate at 16.8% and the greatest risk of mortality (OR: 4.613, 95% CI: 4.132–5.515). For patients in the “Kidney‐metabolic” pattern, the mortality rate was 4.0%, with their mortality risk being 1.571 times higher compared to those without this pattern (OR: 1.571, 95% CI: 1.437–1.717) (Figure 4A).

Logistic analysis in total participants stratified by age revealed that the mortality odds ratios (ORs) for the “Kidney‐metabolic” pattern across the age groups of 60–69, 70–79, 80–89, and ≥ 90 were 1.1 (95% CI: 0.872, 1.388), 1.3 (95% CI: 1.094, 1.545), 1.939 (95% CI: 1.267, 2.226), and 1.614 (95% CI: 1.267, 2.056), respectively (Figure 4B). This indicates an age‐related increase in mortality risk for the “Kidney‐metabolic” pattern, a trend not observed in the other three patterns. Additionally, the trend test results indicated a significant age‐related increase in mortality risk among patients with the “Kidney‐metabolic” pattern (*P* for trend < 0.001). When compared to the 60–69 year‐old control group, OR values for mortality risk in age groups of 70–79, 80–89, and ≥ 90 years were 2.411 (95% CI: 1.863–3.120), 11.469 (95% CI: 9.066–14.508) and 49.237 (95% CI: 37.455–64.724) respectively (Figure 4C).

## 5 Discussion

To the best of our knowledge, this study is the first to utilize inpatient data from comprehensive hospitals to identify the “Kidney‐metabolic” multimorbidity pattern and to its impact on mortality. This approach highlights the significant association between kidney disease and other metabolic disorders in elderly inpatients, particularly in the oldest segment. Understanding this multimorbidity pattern helps us to apply comprehensive intervention including nutritional screening and metabolic risk prevention, and further to optimize strategies of public health among the elderly in China.

The prevalence of multimorbidity ranged from 3.5% to 100% for elderly adults around the world (Hu et al. 2015). We discovered that 38.8% of elderly inpatients were afflicted with multiple chronic diseases. The difference of the multimorbidity prevalence could be attributable to variations in disease selection, demographic features of the samples, and the methodologies employed (Hu et al. 2015; Stirland et al. 2020; Yao et al. 2020). Multimorbidity has a positive correlation with poor health and mortality (Ioakeim‐Skoufa et al. 2020; Jani et al. 2019; Nunes, Flores, et al. 2016). We found that the mortality risk was 2–15 times higher compared to individuals without multimorbidity, in alignment with findings from other studies. Chronic diseases often cluster together either randomly or through certain common pathways, leading to some distinct patterns of multimorbidity. In our study, we discovered that nearly 80% of patients with CKD presented with additional morbidities, indicating a significant contribution of CKD to multimorbidity Utilizing exploratory factor analysis, four multimorbidity patterns were identified named as “Kidney‐metabolic”, “Cerebro‐vascular”, “Cardio‐pulmonary”, and “Thyroid‐digestive” pattern, which respectively reflect the clustering of chronic diseases among the study participants. Notably the “Kidney‐metabolic” pattern, characterized by CKD, Hypertension, Diabetes, and Lipid disorders, exhibited the highest prevalence at 13.3%. The same pattern was rarely reported in elderly adults. Metabolic‐related diseases often formed the multimorbidity pattern with heart and cerebrovascular diseases (Wang et al. 2015; Yao et al. 2020) in previous studies. In our study, they tended to cluster together with kidney disease and form the “Kidney‐metabolic” pattern, rather than with cardiovascular diseases, which highlighted a close relationship between CKD and metabolic‐related diseases.

Since the dawn of nephrology, dietary intervention has been one of the cornerstones of therapeutic intervention, used by nephrologists in an attempt to reduce the symptoms and metabolic complications that characterize CKD and, possibly, to stop its evolution (Messa 2022). Elderly patients with CKD are a vulnerable group for nutritional management, which are at high risk of malnutrition and sarcopenia along with declining kidney function, inflammatory conditions and dietary restrictions (Ertuglu and Ikizler 2024). In the last decade there has been a paradigm shift in the nutritional management of CKD (Hu et al. 2021). Appropriate nutritional management has evolved from merely protecting the kidneys to systematically maintaining the nutritional and metabolic homeostasis throughout the body and should be embedded in the overall lifestyle patterns. The components of metabolic syndrome (central obesity, hypertension, hyperglycemia, and dyslipidemia) accelerate kidney damage and systemic metabolic disorders in synergy with CKD through common pathways such as insulin resistance, obesity, inflammation, oxidative stress, and endothelial dysfunction, which lead to complex metabolic risk conditions (Ma et al. 2018; Yang et al. 2020). Unhealthy lifestyle characterized by high salt, high refined carbohydrates, low dietary fiber and low physical activity is significantly associated with the burden of metabolic health (Ertuglu and Ikizler 2024).

The multimorbidity pattern we found partly aligns with the recent Cardiovascular‐Kidney‐Metabolic (CKM) framework proposed by the American Heart Association (Ndumele et al. 2023). The concept of CKM placed even greater emphasized on the impact of “kidney‐metabolic” on cardiovascular events. Our “Kidney‐metabolic” multimorbidity pattern further highlighted some certain cluster of CKD and metabolic disorders. CKM focused on the risk stratification of the entire population, with the ultimate goal of preventing cardiovascular events by controlling risk factors early. Our research focused on the elderly inpatients and employed advanced statistical methods to analyze the mutlimorbidity pattern of Kidney‐metabolic, which was highly prevalent and was closely related to mortality. In our study, four multimorbidity patterns we found were all positively associated with an increased risk of mortality. However, the impact of the “Kidney‐metabolic” pattern on mortality was found to significantly increase with age, a trend not observed in the other three patterns. The mortality risk for patients aged over 80 years with the “Kidney‐metabolic” pattern was 11–49 times higher than for those aged 60–69 years, underscoring the substantial impact of this pattern on the health and lifespan. It reminded us multiple nutrition‐metabolic disorders may be the important cause of accelerating death in the elderly inpatients, particularly in the oldest demographic. CKD is considered to be an important metabolic and nutrition‐related chronic disease. Previous researches have shown that among the elderly population (over 85 years old), renal dysfunction is a stronger predictor of all‐cause mortality and re‐hospitalization compared to heart failure or myocardial infarction (Cavanaugh et al. 2017). Many cardiovascular events have directly triggered by electrolyte imbalances or volume overload, which is often the result of renal dysfunction. The essence of “Kidney‐metabolic” pattern is a complex of nutritional metabolism, which directly erodes the nutritional reserves and balance of the elderly. They may serve as “amplifiers” and “accelerators” that determine the course of the disease, trigger acute events, and affect the overall functional status. It becomes more prominent among the elderly population and increased by age. Therefore, transitioning from traditional single‐disease treatment to the maintenance of overall homeostasis and prioritizing assessment and meticulous management of kidney‐metabolic disorders are crucial for metabolic health and survival.

The overlay of China's aging population and burden of multiple chronic diseases post great severe challenges currently to the public health (Di Renzo et al. 2021). Especially, the “Kideny‐metabolic” pattern requires us to elevate “integrated medical nutrition therapy” (MNT) in the elderly to the core position of the management of multimorbidity and run through the entire progress in the elderly population. Previous observational studies suggested that dietary patterns that promote metabolic health, such as diets based on eating vegetables, nuts, legumes, whole grains, and fish and poultry, with less red meat and fewer processed foods, are associated with reduced risk of cardiovascular events and mortality (Messa 2022; Yee‐Moon Wang et al. 2023). The traditional model of single “low‐protein diet” or “diabetic diet” is no longer applicable (Lachowicz‐Wisniewska and Kotowska 2025). For patients with “Kideny‐metabolic” pattern, the need of “MNT” should be emphasized, which is controlling the quality and total amount of carbohydrates and sodium salts, carefully selecting protein sources (giving priority to high biological value proteins) and optimizing fatty acid composition (such as increasing the ratio of monounsaturated and omega‐3 fatty acids) so as to synergistically manage blood pressure, blood glucose, lipid and urine protein levels.

However, in China, precisely in the crucial aspect of “MNT”, we have some huge practical shortcomings until now (Meng 2022). First, the improvement of residents unhealthy dietary habits (such as high salt, high oil, high sugar, low intake of whole grains and low fruit intake) had been progressing slowly in China. Second, for elderly patients with multimorbidity, there was a lack of standard pathways, professional teams and systematic support in formulating and implementing safe, effective, individualized and sustainable integrated nutrition management. Studying the characteristics of multimorbidity pattern and their impacts on mortality will contribute to the establishment and implementation of the geriatric nutrition and chronic disease management system so as to improve public health.

Our study possesses several limitations that merit discussion. These limitations are essential to consider when interpreting the findings and planning future research directions. First, this was a single‐center study conducted at a tertiary comprehensive hospital. Our Elderly Multimorbidity Database included a substantial number of inpatients from a wide range of provinces nationwide, demonstrating broad representativeness of this demographic. However, the majority of patients were from eastern China. Given potential significant geographic variations in patient characteristics, the generalizability of the study population may be limited. Second, chronic diseases were identified through electronic medical records, which may lead to underestimated diagnoses. In the factor analysis, we excluded certain types of cancer and other chronic diseases with relatively low prevalence. These might led to some statistical biases. Third, Our research lacked longitudinal data, especially data on nutrition intervention. In the future, we will further design and conduct the studies.

In conclusion, multimorbidity was common and positively correlated with a poor health and mortality in elderly patients. “Kidney‐metabolic” pattern was found to be the most prevalent pattern, which highlighted intertwined nutrition and metabolic‐related conditions and represented predominant combined risk factors for metabolic health. Recognizing such pattern helps to support early metabolic risk screening, integrated medical nutrition therapy, and comprehensive multimorbidity management so as to reduce the burden of chronic diseases and improve healthy aging.

## Author Contributions

Conception/design of the work: Jing Chen, Huaizhou You, Lan Xu. Study oversight: Jing chen, Huaizhou You, Guoyou Qin. Data Acquisition: Qiang Shao, Hong Huang. Data Analysis: Guoyou Qin, Mengjing Wang, Lan Xu. Drafting of the manuscript: Lan Xu, Huaizhou You, Mengjing Wang. All authors were involved in data interpretation and review of the final manuscript.

## Funding

This work was supported by grants from the National Key R&D Program of China (2021YFC2500202, 2020YFC2005000), Shanghai Municipal Health Commission (GWVI‐11.1‐27, 202140038), Innovation action plan project of Shanghai Science and Technology Commission (No. 20Y11904500, 21ZR1411100, 21Y11904200), Zhejiang Provincial Natural Science Foundation of China (Q21H050026), and the Zhejiang Provincial Medical and Health Research Project (2021RC067).

## Ethics Statement

The study adhered to the Declaration of Helsinki and received approval from the local Medical Ethics Committee at Huashan Hospital, Fudan University, Shanghai, China (Approval number: KY2019‐362).

## Conflicts of Interest

The authors declare no conflicts of interest.

## Supporting information


**Figure S1:** Participant flow diagram.
**Figure S2:** Map of provincial regions in the survey.
**Table S1:** The number and percentages in total participants affected by each chronic disease.
**Table S2:** The distribution of chronic diseases in participants with multimorbidity.
**Table S3:** Geographical distribution of total participants.
**Table S4:** The distribution of chronic diseases in participants with chronic kidney disease.

## Data Availability

The data that support the findings of this study are available from the corresponding author upon reasonable request.
