# Supplementary material for: Relevance of Kidney‐Metabolic Multimorbidity Pattern to Metabolic Health and Mortality Among Elderly Inpatients in China
Source: Food Sci Nutr. 2026 Mar 23;14(3):e71612. doi: 10.1002/fsn3.71612 (PMC13093273; doi:10.1002/fsn3.71612)
Supplement: Supplementary file 1 — Figure S1: Participant flow diagram. Figure S2: Map of provincial regions in the survey. Table S1: The number and percentages in total participants affected by each chronic disease. Table S2: The distribution of chronic diseases in participants with multimorbidity. Table S3: Geographical distribution of total participants. Table S4: The distribution of chronic diseases in participants with chronic kidney disease. [file FSN3-14-e71612-s001.doc]

**Caption for supplementary material**

**Supplemental Figure S1. Participant flow diagram**

**Supplemental Figure S2. Map of provincial regions in the survey**

**Supplemental Table S1. The number and percentages in total participants affected by each chronic disease**

**Supplemental Table S2. The distribution of chronic diseases in participants with multimorbidity**

**Supplemental Table S3. Geographical distribution of total participants**

**Supplemental Table S4. The distribution of chronic diseases in participants with chronic kidney disease**

**Supplemental Figure S1. Participant flow diagram**

All inpatient database (N=557942)

2013.1.24-2019.1.11

N=557942

Excluded (N=368326)

Age<60 years old

Elderly inpatient database

(N=189616)

N=189616

Excluded (N=25990)

non-gender (N=24)

non-outcomes (N=10)

diagnoses beyond the range of 48 diseases (N=25956)

Elderly multimorbidity database

(N=163626)

**Supplemental Figure S2.Map of provincial regions in the survey**


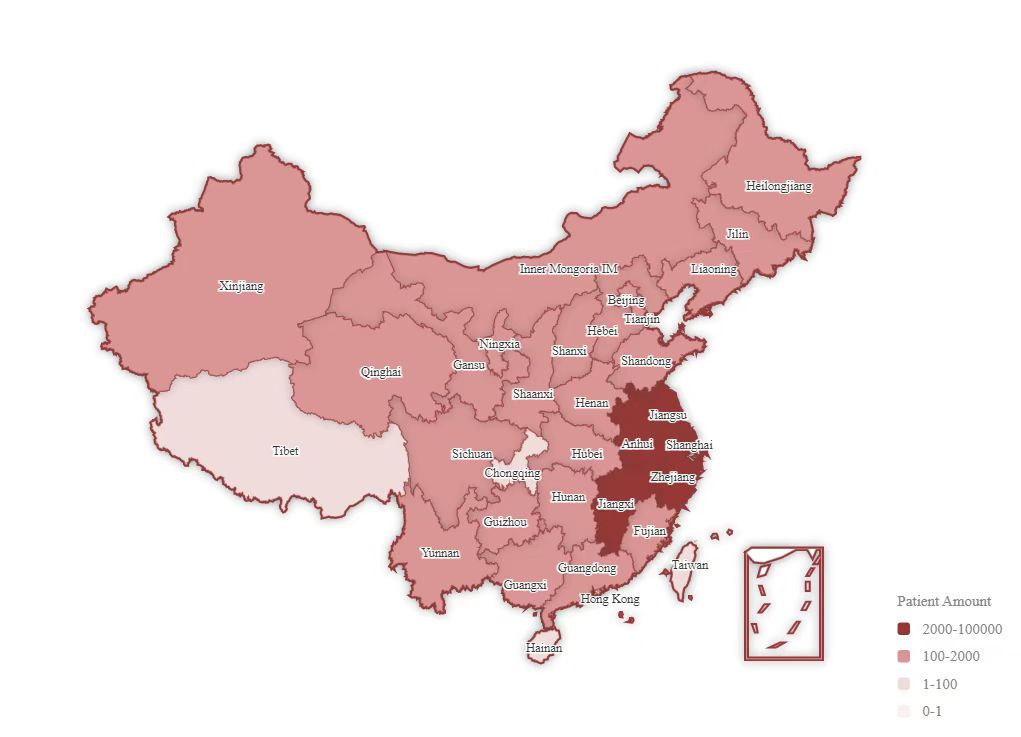


**Supplemental Table S1. The number and percentages in total participants affected by each chronic disease.**

| Chronic disease | Overall, n(%) | Female, n(%) | Male, n(%) |
| --- | --- | --- | --- |
| All patients | 163626(100) | 73607(44.98) | 90019(55.02) |
| Cancer | 54022(33.02) | 23248(43.03) | 30774(56.96) |
| Hypertension | 37763(23.07) | 16316(43.20) | 21447(56.79) |
| Diabetes | 19870(12.14) | 8666(43.61) | 11204(56.38) |
| Stroke/cerebrovascular disease | 17849(10.90) | 6832(38.27) | 11017(61.72) |
| Ischemic heart disease | 16128(9.85) | 6350(39.37) | 9778(60.62) |
| Cataract/glaucoma | 13361(8.16) | 7613(56.97) | 5748(43.02) |
| Osteoarthropathy | 11740(7.17) | 7332(62.45) | 4408(37.54) |
| Chronic kidney disease | 9824(6.00) | 3884(39.53) | 5940(60.46) |
| Biliary disease | 9793(5.98) | 4829(49.31) | 4964(50.68) |
| Gastropathy/esophagus disease | 7175(4.38) | 3083(42.96) | 4092(57.03) |
| Peripheral vascular disease | 7050(4.31) | 2506(35.54) | 4544(64.45) |
| Chronic liver disease | 6310(3.86) | 2837(44.96) | 3473(55.03) |
| Hernia | 5827(3.56) | 890(15.27) | 4937(84.72) |
| Other cardiac arrhythmias | 5763(3.52) | 2469(42.84) | 3294(57.15) |
| Thyroid disease | 4525(2.76) | 2724(60.19) | 1801(39.80) |
| Chronic bronchitis/COPD | 4004(2.45) | 893(22.30) | 3111(77.69) |
| Prostate disease | 3938(2.41) | 4(0.101) | 3934(99.89) |
| Connective tissue disease | 3752(2.29) | 2271(60.52) | 1481(39.47) |
| Psoriasis/eczema | 3686(2.25) | 1437(38.98) | 2249(61.01) |
| Gallbladder/ureteral disease | 3419(2.09) | 1075(31.44) | 2344(68.55) |
| Chronic heart failure | 3217(1.97) | 1303(40.50) | 1914(59.49) |
| Benign tumour | 2966(1.81) | 1757(59.23) | 1209(40.76) |
| Blindness/low vision | 2785(1.70) | 1431(51.38) | 1354(48.61) |
| Lipid disorder | 2784(1.70) | 1636(58.76) | 1148(41.23) |
| Atrial flutter/atrial fibrillation | 2693(1.65) | 1105(41.03) | 1588(58.96) |
| Viral hepatitis | 2274(1.39) | 790(34.74) | 1484(65.25) |
| Pituitary disease | 2224(1.36) | 963(43.30) | 1261(56.69) |
| Peripheral neuropathy | 2211(1.35) | 972(43.96) | 1239(56.03) |
| Muscle/tendon disease | 2100(1.28) | 1211(57.66) | 889(42.33) |
| Neurological disease | 1972(1.21) | 864(43.81) | 1108(56.18) |
| Hyperuricemia/gout | 1597(0.98) | 283(17.72) | 1314(82.27) |
| Other heart disease | 1588(0.97) | 638(40.17) | 950(59.82) |
| Anemia | 1349(0.82) | 577(42.77) | 772(57.22) |
| Spine/intervertebral disc disease | 1262(0.77) | 616(48.81) | 646(51.18) |
| Osteoporosis | 1205(0.74) | 830(68.87) | 375(31.12) |
| Asthma | 966(0.59) | 454(46.99) | 512(53.00) |
| Parkinson's disease | 922(0.56) | 335(36.33) | 587(63.66) |
| Epilepsy | 685(0.42) | 234(34.16) | 451(65.83) |
| Chronic pharyngitis/laryngitis/tonsillitis | 678(0.41) | 223(32.89) | 455(67.10) |
| Paranasal sinus disease | 668(0.41) | 267(39.97) | 401(60.02) |
| Bronchiectasis | 584(0.36) | 284(48.63) | 300(51.36) |
| Cognitive impairment | 581(0.36) | 242(41.65) | 339(58.34) |
| Anxiety/depression | 515(0.31) | 273(53.00) | 242(46.99) |
| Hearing loss/tinnitus | 410(0.25) | 190(46.34) | 220(53.65) |
| Chronic painful condition | 354(0.22) | 162(45.76) | 192(54.23) |
| Irritable bowel syndrome | 269(0.16) | 110(40.89) | 159(59.10) |
| Inflammatory bowel disease | 231(0.14) | 117(50.64) | 114(49.35) |
| Diverticular disease of intestine | 143(0.09) | 67(46.85) | 76(53.14) |

COPD, Chronic obstructive pulmonary disease

**Supplemental Table S2. The distribution of chronic diseases in participants with multimorbidity**

| Chronic diseases | With other diseases, n(%) | with 1 other disease, n(%) | with 2 other diseases, n(%) | with 3 or more other diseases, n(%) |
| --- | --- | --- | --- | --- |
| All patients | 63477 | 33256 | 16715 | 13506 |
| Hypertension | 35612(56.10) | 13169(39.60) | 11676(69.85) | 10767(79.72) |
| Diabetes | 18877(29.74) | 4836(14.54) | 6799(40.68) | 7242(53.62) |
| Cancer | 15650(24.65) | 8657(26.03) | 3872(23.16) | 3121(23.11) |
| Ischemic heart disease | 13986(21.89) | 4310(12.96) | 3742(22.39) | 5844(43.27) |
| Stroke/cerebrovascular disease | 11741(18.50) | 3900(11.73) | 3223(19.28) | 4618(34.19) |
| Chronic kidney disease | 7926(12.49) | 2526(7.60) | 2162(12.93) | 3238(23.97) |
| Biliary disease | 5973(9.41) | 2395(7.20) | 1300(7.78) | 2278(16.87) |
| Gastropathy/esophagus disease | 5456(8.60) | 1702(5.12) | 1295(7.75) | 2459(18.21) |
| Chronic liver disease | 5449(8.58) | 1592(4.79) | 1407(8.42) | 2450(18.14) |
| Cataract/glaucoma | 5414(8.53) | 2711(8.15) | 1267(7.58) | 1436(10.63) |
| Other cardiac arrhythmias | 5227(8.23) | 1403(4.22) | 1270(7.60) | 2554(18.91) |
| Peripheral vascular disease | 4224(6.65) | 1480(4.45) | 1017(6.08) | 1727(12.79) |
| Chronic Bronchitis/COPD | 3516(5.54) | 913(2.75) | 820(4.91) | 1783(13.20) |
| Thyroid disease | 3221(5.07) | 756(2.27) | 640(3.83) | 1825(13.51) |
| Chronic heart failure | 3136(4.94) | 597(1.80) | 682(4.08) | 1857(13.75) |
| Prostate disease | 3092(4.87) | 883(2.66) | 616(3.69) | 1593(11.79) |
| Osteoarthropathy | 3062(4.82) | 1845(5.55) | 565(3.38) | 652(4.83) |
| Lipid disorder | 2702(4.26) | 483(1.45) | 737(4.41) | 1482(10.97) |
| Atrial flutter/atrial fibrillation | 2625(4.14) | 539(1.62) | 687(4.11) | 1399(10.36) |
| Connective tissue disease | 2456(3.87) | 949(2.85) | 713(4.27) | 794(5.88) |
| Blindness/Low vision | 2386(3.76) | 1339(4.03) | 603(3.61) | 444(3.29) |
| Gallbladder/ureteral disease | 2095(3.30) | 1249(3.76) | 394(2.36) | 452(3.35) |
| Viral Hepatitis | 1802(2.84) | 701(2.11) | 543(3.25) | 558(4.13) |
| Benign tumour | 1773(2.79) | 1293(3.89) | 232(1.39) | 248(1.84) |
| Hernia | 1639(2.58) | 770(2.32) | 405(2.42) | 464(3.44) |
| Hyperuricemia/gout | 1533(2.42) | 175(0.53) | 382(2.29) | 976(7.23) |
| Other heart disease | 1402(2.21) | 392(1.18) | 383(2.29) | 627(4.64) |
| Anemia | 1237(1.95) | 307(0.92) | 291(1.74) | 639(4.73) |
| Neurological disease | 1181(1.86) | 553(1.66) | 273(1.63) | 355(2.63) |
| Osteoporosis | 1162(1.83) | 121(0.36) | 197(1.18) | 844(6.25) |
| Psoriasis/eczema | 1007(1.59%) | 444(1.34) | 232(1.39) | 331(2.45) |
| Muscle/tendon disease | 996(1.57) | 709(2.13) | 137(0.82) | 150(1.11) |
| Peripheral neuropathy | 890(1.47) | 567(1.70) | 184(1.10) | 139(1.03) |
| Asthma | 810(1.28%) | 253(0.76) | 226(1.35) | 331(2.45) |
| Parkinson's disease | 726(1.14) | 214(0.64) | 176(1.05) | 336(2.49) |
| Pituitary disease | 720(1.13) | 363(1.09) | 136(0.81) | 221(1.64) |
| Spine/intervertebral disc disease | 677(1.07) | 403(1.21) | 101(0.60) | 173(1.28) |
| Cognitive impairment | 520(0.82) | 107(0.32) | 97(0.58) | 316(2.34) |
| Epilepsy | 516(0.81) | 160(0.48) | 139(0.83) | 217(1.61) |
| Anxiety/depression | 475(0.75) | 88(0.26) | 102(0.61) | 285(2.11) |
| Bronchiectasis | 456(0.72) | 134(0.40) | 122(0.73) | 200(1.48) |
| Chronic pharyngitis/laryngitis/tonsillitis | 364(0.57) | 125(0.38) | 66(0.39) | 173(1.28) |
| Paranasal sinus disease | 269(0.42) | 93(0.28） | 51(0.31) | 125(0.93) |
| Chronic painful condition | 245(0.39) | 64(0.19) | 37(0.22) | 144(1.07) |
| Hearing loss/tinnitus | 244(0.38) | 85(0.26) | 55(0.33) | 104(0.77) |
| Irritable bowel syndrome | 223(0.35) | 56(0.17) | 26(0.16) | 141(1.04) |
| Inflammatory bowel disease | 161(0.25) | 63(0.19) | 36(0.22) | 62(0.46) |
| Diverticular disease of intestine | 119(0.19) | 38(0.11) | 29(0.17) | 52(0.39) |

COPD, Chronic obstructive pulmonary disease

*Disease with grey shading were selected into the exploratory factor analysis

**Supplemental Table S3. Geographical distribution of total participants**

| Province | Record | Percentage |
| --- | --- | --- |
| All patients | 163626 | 100% |
| Shanghai | 104236 | 63.70% |
| Jiangsu | 18421 | 11.26% |
| Zhejiang | 16765 | 10.25% |
| Anhui | 7025 | 4.29% |
| Jiangxi | 3723 | 2.27% |
| Fujian | 1462 | 0.90% |
| Helongjiang | 1212 | 0.74% |
| Shandong | 1181 | 0.72% |
| Henan | 1145 | 0.70% |
| Xinjiang | 1016 | 0.62% |
| Hubei | 894 | 0.55% |
| Sichuan | 834 | 0.51% |
| Gansu | 627 | 0.38% |
| Liaoning | 589 | 0.36% |
| Guizhou | 566 | 0.34% |
| Jilin | 533 | 0.32% |
| Hunan | 489 | 0.30% |
| Shanxi | 462 | 0.28% |
| Hebei | 386 | 0.24% |
| Shanxi | 329 | 0.20% |
| Beijing | 301 | 0.18% |
| Yunnan | 263 | 0.16% |
| Inner Mongiria | 254 | 0.15% |
| Guangdong | 211 | 0.13% |
| Qinghai | 176 | 0.11% |
| Guangxi | 162 | 0.10% |
| Ninxia | 158 | 0.10% |
| Tianjin | 141 | 0.08% |
| Hainan | 30 | 0.02% |
| Xizang | 15 | 0.01% |
| Taiwan | 10 | 0.01% |
| Chongqing | 6 | 0.01% |
| Hong Kong | 2 | 0.01% |

**Supplemental Table S4. The distribution of chronic diseases in participants with chronic kidney disease**

|  | CKD with 1 other disease, n(%) | CKD with 2 other diseases, n(%) | CKD with 3 or more other diseases, n(%) |
| --- | --- | --- | --- |
| All patients | 2526 | 2162 | 3274 |
| Hypertension | 712(28.19) | 1389(64.25) | 2545(77.73) |
| Diabetes | 212(8.39) | 717(33.16) | 1612(49.24) |
| Ischemic heart disease | 48(1.90) | 202(9.34) | 1161(35.46) |
| Stroke/cerebrovascular disease | 33(1.31) | 126(5.83) | 853(26.05) |
| Chronic liver disease | 72(2.85) | 140(6.48) | 720(21.99) |
| Cancer | 337(13.34) | 319(14.75) | 627(19.15) |
| Biliary disease | 72(2.85) | 124(5.74) | 603(18.42) |
| Gastropathy/esophagus disease | 36(1.43) | 83(3.84) | 576(17.59) |
| Chronic heart failure | 24(0.95) | 78(3.61) | 575(17.56) |
| Thyroid disorders | 20(0.79) | 48(2.22) | 521(15.91) |
| Other cardiac arrhythmias | 17(0.67) | 55(2.54) | 491(15) |
| Hyperuricemia/gout | 38(1.50) | 134(6.20) | 432(13.19) |
| Peripheral vascular disease | 38(1.50) | 57(2.64) | 421(12.86) |
| Chronic bronchitis/COPD | 23(0.91) | 61(2.82) | 385(11.76) |
| Prostate disease | 73(2.89) | 93(4.30) | 367(11.21) |
| Cataract/glaucoma | 17(0.67) | 36(1.67) | 298(9.10) |
| Lipid disorder | 10(0.40) | 20(0.93) | 295(9.01) |
| Atrial flutter/atrial fibrillation | 5(0.20) | 27(1.25) | 262(8.00) |
| Anemia | 19(0.75) | 56(2.59) | 241(7.36) |
| Gallbladder/ureteral disease | 0(0) | 203(9.39) | 219(6.69) |
| Osteoporosis | 4(0.16) | 13(0.60) | 204(6.23) |
| Connective tissue disease | 57(2.26) | 100(4.63) | 202(6.17) |
| Viral Hepatitis | 14(0.55) | 28(1.30) | 124(3.79) |
| Osteoarthropathy | 15(0.59) | 35(1.62) | 124(3.79) |
| Other heart disease | 18(0.71) | 15(0.69) | 120(3.67) |
| Hernia | 16(0.63) | 26(1.20) | 93(2.84) |
| Psoriasis/eczema | 18(0.71) | 30(1.39) | 79(2.41) |
| Blindness/Low vision | 0(0) | 3(0.14) | 62(1.89) |
| Parkinson's disease | 4(0.16) | 9(0.42) | 60(1.83) |
| Anxiety/depression | 0(0) | 4(0.19) | 57(1.74) |
| Benign tumour | 14(0.55） | 10(0.46) | 54(1.65) |
| Asthma | 2(0.08) | 11(0.51) | 53(1.62) |
| Neurological disorders | 5(0.20) | 11(0.51) | 53(1.62) |
| Cognitive impairment | 2(0.08) | 3(0.14) | 53(1.62) |
| Pituitary disease | 3(0.12) | 9(0.42) | 52(1.59) |
| Epilepsy | 3(0.12) | 5(0.23) | 46(1.41) |
| Bronchiectasis | 1(0.04) | 7(0.32) | 43(1.31) |
| Irritable bowel syndrome | 2(0.08) | 1(0.05) | 35(1.07) |
| Spine/intervertebral disc disease | 1(0.04) | 4(0.19) | 35(1.07) |
| Chronic pharyngitis/laryngitis/tonsillitis | 3(0.12) | 3(0.14) | 34(1.04) |
| Muscle/tendon disease | 2(0.08) | 11(0.51) | 33(1.01) |
| Chronic painful condition | 1(0.04) | 2(0.09) | 28(0.86) |
| Hearing loss/tinnitus | 3(0.12) | 4(0.19) | 25(0.76) |
| Paranasal sinus disease | 2(0.08) | 2(0.09) | 23(0.70) |
| Peripheral neuropathy | 0(0) | 6(0.28) | 22(0.67) |
| Inflammatory bowel disease | 0(0) | 1(0.05) | 12(0.37) |
| Diverticular disease of intestine | 1(0.04) | 3(0.14) | 9(0.27) |

COPD, Chronic obstructive pulmonary disease; CKD,Chronic kidney disease
